# Delayed-onset interface fluid syndrome after LASIK following phacotrabeculectomy

**DOI:** 10.1186/s12886-019-1077-2

**Published:** 2019-03-12

**Authors:** Chung Young Kim, Young Ho Jung, Eun Ji Lee, Joon Young Hyon, Kyu Hyung Park, Tae Woo Kim

**Affiliations:** 1 Department of Ophthalmology, Seoul National University Hospital, Seoul, South Korea; 2 2nd Air Defense Missile Brigade, Republic of Korea Air Force, Gapyeong, South Korea; 3 Department of Ophthalmology, Seoul National University Bundang Hospital, 300, Gumi-dong, Bundang-gu, Seongnam, Gyeonggi-do 13620 South Korea

**Keywords:** Interface fluid syndrome, LASIK, Glaucoma, Phacotrabeculectomy

## Abstract

**Background:**

Interface fluid syndrome (IFS) is an unusual complication after laser-assisted in-situ keratomileusis (LASIK). We report the first case of IFS after uncomplicated phacotrabeculectomy in a patient who had undergone LASIK 10 years previously. This case emphasizes the importance of intraocular pressure (IOP) interpretation in eyes that have undergone LASIK.

**Case presentation:**

A 30-year-old woman with a history of LASIK surgery presented to glaucoma clinic due to uncontrolled IOP despite of maximally tolerable medical treatment. After receiving phacotrabeculectomy, IOP decreased to 3 mmHg on the first postoperative day, but again increased up to 21 mmHg and a diffuse corneal edema with cloudy flap interface was demonstrated by slit-lamp microscopy. Corneal edema was sustained even after the IOP was lowered to 14 mmHg. Spectral-domain optical coherence tomography scanning of the cornea revealed a diffuse, thin fluid pocket in the corneal interface. After laser lysis of the scleral flap sutures, IOP was further decreased to 9 mmHg and interface fluid was resolved.

**Conclusion:**

IFS should be considered as a possible cause of postoperative corneal edema despite of low IOP in the eyes that underwent LASIK surgery. Additional IOP lowering may be helpful for resolving the corneal edema.

## Background

Interface fluid syndrome (IFS) is an unusual complication after laser-assisted in-situ keratomileusis (LASIK) that is characterized by diffuse fluid accumulation within the flap interface. Although elevation of intraocular pressure (IOP) is the main sign associated with the IFS [1], falsely low IOP readings after LASIK could mimic the condition and delay an accurate diagnosis [2]. We report the first case of IFS after uncomplicated phacotrabeculectomy in a patient who had undergone LASIK 10 years previously. The IFS did not resolve when the IOP was reduced to within the statistically normal range, but it did resolve with further IOP reduction. This case emphasizes the importance of IOP interpretation in eyes that have undergone LASIK.

## Case report

A 30-year-old woman was presented to a glaucoma clinic due to uncontrolled IOP. She had undergone bilateral LASIK 10 years previously, and had been treated with oral and topical steroids as well as albendazole for 10 months for uveitis associated with ocular toxocariasis in the left eye. Sub-Tenon injection of triamcinolone acetonide (40 mg) had also been performed 5 months previously.

At the first visit, her visual acuity was 20/200 and the IOP was 30 mmHg in the left eye measured by Goldmann applanation tonometry (GAT). Slit-lamp examination revealed Grade 1 posterior subcapsular opacity, and fundus examination showed glaucomatous change in the optic nerve head. Inflammatory cells were not detected in either the anterior or posterior chamber. Despite maximally tolerable medical treatment, the IOP subsequently increased up to 32 mmHg, and her visual acuity worsened to 20/500 with ongoing glaucomatous optic nerve damage and progression of posterior subcapsular opacity. Phacotrabeculectomy with topically applied mitomycin-C (0.04%) was then performed.

**Fig. 1:**
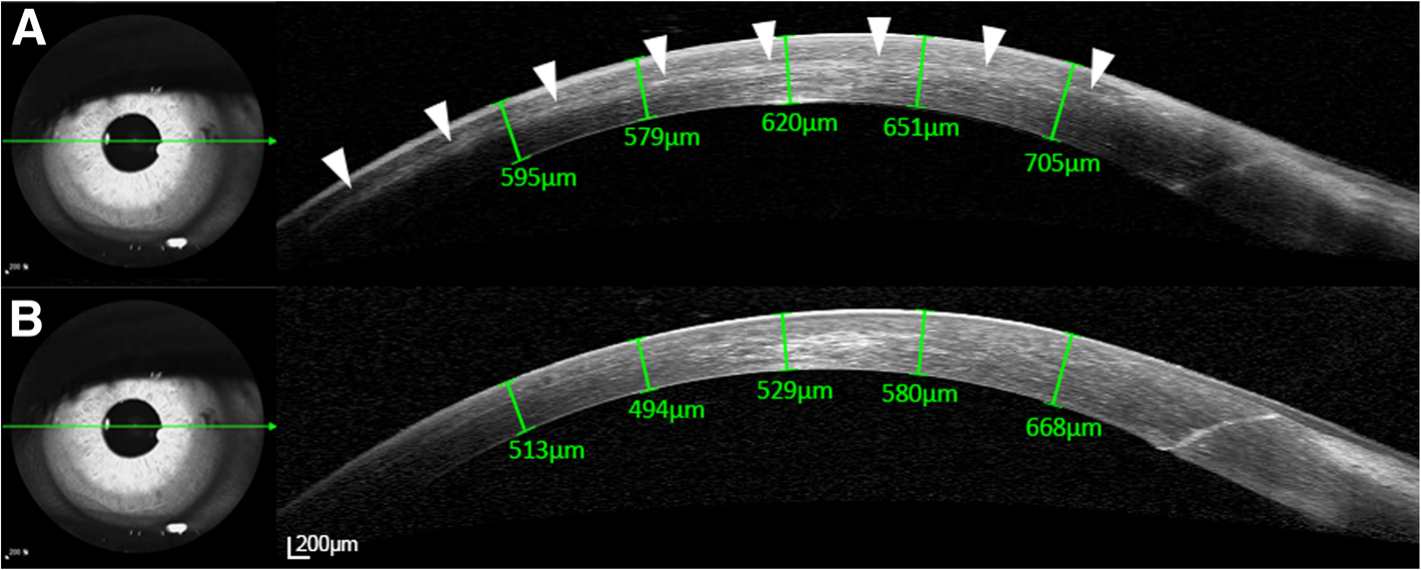
Spectral-domain optical coherence tomography scanning of the cornea before (**a**) and after (**b**) performing suture lysis using an argon laser. Note that the diffuse and thin fluid pocket in the corneal interface region (arrowheads) resolved when the intraocular pressure was lowered from 14 to 9 mmHg

The IOP was 3 mmHg by GAT on the first postoperative day but increased up to 21 mmHg on the following day. Her visual acuity was hand movement and could not be corrected. A diffuse corneal edema with a cloudy flap interface was noted in a slit-lamp examination. At 1 week postoperatively, the IOP had decreased to 14 mmHg after the application of brimonidine (0.2%)/timolol (0.5%) twice daily, but the corneal edema did not resolve. Spectral-domain optical coherence tomography (SD-OCT) scanning revealed a diffuse and thin fluid pocket in the corneal interface region (Fig. 1A). After using an argon laser to perform suture-lysis of the scleral flap on the following day, the IOP decreased to 9 mmHg and the visual acuity improved to 20/150. Resolution of the interface fluid was noted by SD-OCT (Fig. 1B). Central corneal thicknesses were 553.5μm preoperatively and 576.6μm at 14 days postoperatively. Eight months postoperatively, the IOP was maintained at 8 mmHg without using topical IOP-lowering agents, the cornea was clear without any interface haze by detailed slit-lamp examination, and the corrected visual acuity was 20/100.

## Discussions and conclusions

Lowering of IOP commonly results in the resolution of interface fluid that can appear after LASIK [3]. However, the interface fluid in our case did not improve even when the IOP was reduced to within the statistically normal range; instead, a substantial IOP lowering down to a subnormal level was necessary for resolution of this fluid. We speculate that the decreased corneal thickness after LASIK and the ability of the accumulated fluid to absorb shock might have resulted in the underestimation of IOP [4]. This suggests that the target IOP should be lower in eyes that have undergone LASIK to allow for the possibility of falsely low IOP reading. In this case, IOP was measured at central cornea, but IOP measurement peripheral to the LASIK flap is also required for accuracy.

In eyes having a history of LASIK, the possibility of IFS should be considered as a possible cause of postoperative corneal edema even when the IOP is within normal range, particularly when the edema is long-standing and refractory to conventional treatment. An additional IOP lowering beyond the normal range may be helpful for resolving the corneal edema.

## Abbreviations

GAT: Goldmann applanation tonometry
IFS: Interface fluid syndrome
IOP: Intraocular pressure
LASIK: laser-assisted in-situ keratomileusis
SD-OCT: Spectral-domain optical coherence tomography
